# High-Resolution NMR Reveals Secondary Structure and Folding of Amino Acid Transporter from Outer Chloroplast Membrane

**DOI:** 10.1371/journal.pone.0078116

**Published:** 2013-10-29

**Authors:** James D. Zook, Trivikram R. Molugu, Neil E. Jacobsen, Guangxin Lin, Jürgen Soll, Brian R. Cherry, Michael F. Brown, Petra Fromme

**Affiliations:** 1 Department of Chemistry and Biochemistry, Arizona State University, Tempe, Arizona, United States of America; 2 Department of Chemistry and Biochemistry, University of Arizona, Tucson, Arizona, United States of America; 3 Saudi Basic Industries Corporation, Mount Vernon, Indiana, United States of America; 4 Department of Biology, Ludwig-Maximilians-University, München, Germany; 5 Department of Physics, University of Arizona, Tucson, Arizona, United States of America; University of Saskatchewan, Canada

## Abstract

Solving high-resolution structures for membrane proteins continues to be a daunting challenge in the structural biology community. In this study we report our high-resolution NMR results for a transmembrane protein, outer envelope protein of molar mass 16 kDa (OEP16), an amino acid transporter from the outer membrane of chloroplasts. Three-dimensional, high-resolution NMR experiments on the ^13^C, ^15^N, ^2^H-triply-labeled protein were used to assign protein backbone resonances and to obtain secondary structure information. The results yield over 95% assignment of N, H_N_, CO, C_α_, and C_β_ chemical shifts, which is essential for obtaining a high resolution structure from NMR data. Chemical shift analysis from the assignment data reveals experimental evidence for the first time on the location of the secondary structure elements on a per residue basis. In addition *T*_1*Z*_ and *T_2_* relaxation experiments were performed in order to better understand the protein dynamics. Arginine titration experiments yield an insight into the amino acid residues responsible for protein transporter function. The results provide the necessary basis for high-resolution structural determination of this important plant membrane protein.

## Introduction

Integral membrane proteins are a rapidly growing field of interest in structural biology and biochemistry. They are responsible for a plethora of cell functions, ranging from energy generation by enzymes involved in respiration and photosynthesis such as ATP synthase [1], to cell signaling as shown by the large number of integral membrane receptors [2]. However, structure determination of these proteins remains a formidable challenge. While most of the 300 structures of membrane proteins solved so far are determined by X-ray diffraction, crystallization is difficult and is a major bottleneck for solving membrane structures [3], [4]. Improvements in magnetic resonance technology provide new methods as a powerful tool for in-depth analysis of the structure and function of membrane proteins. Although nuclear magnetic resonance (NMR) spectroscopy shows great promise in the field of structure determination, it remains a challenge for membrane proteins for several reasons. One such hurdle involves obtaining high yields of isotope-enriched proteins that are structurally stable in detergent micelles at concentrations high enough to produce adequate signal to noise. A further challenge is specific to proteins that are largely α-helical (as seen by many transmembrane proteins), which display spectra that have a narrow chemical shift dispersion in the ^1^H dimension. This narrow ^1^H dispersion, combined with the increased number of residues present in larger membrane proteins, yields a problem with regard to peak overlap in high-resolution NMR spectra. Yet another obstacle to consider is the necessity of solubilizing the protein in detergent micelles, which are used to maintain protein structural integrity. This increase in size of the complex results in slower rotational averaging, and therefore decreased transverse relaxation times [5].

One integral membrane protein that has shown promise as a target for NMR study is the outer envelope protein with a molecular mass of 16 kDa (OEP16) from the chloroplast membrane [6]. OEP16 is a transmembrane (TM) protein that shares some sequence homologies (52%) to a putative protein from the mitochondrial membrane translocase of the inner membrane (TIM) that may be part of the protein translocase complex (UniProtKB Accession number: ABF95523.1). Notably, OEP16 is located within the outer membrane of chloroplasts, and forms a channel for selective diffusion of amino acids into the intermembrane space. This pore-forming protein is remarkably selective, and may supply the chloroplast organelle with the amino acids for use in protein expression [7]. The first structural information for OEP16 was based on hydropathy plots from the amino acid sequence and circular dichroism (CD) experiments in phosphatidylcholine liposomes [7]. These studies hypothesized that OEP16 may consist of three TM helices with the N-terminus of the protein forming a β-sheet [7], [8]. A later model suggested a four TM-helix bundle [9], which contrasts with the textbook view that nearly all outer membrane channels form β-barrels. Yet improved CD spectral data support the four-helix bundle hypothesis [6]. Dimers have been considered from cross-linking studies [7]; electron micrographs have led to the suggestion of trimer formation [10]; and moreover hexameric and higher oligomeric forms have been hypothesized from gel filtration data [6].

This paper reports the results of NMR experiments for OEP16 in sodium dodecyl sulfate (SDS) detergent micelles. High yields of recombinantly expressed ^15^N-enriched OEP16 in minimal media have been previously reported [6]. These results give a starting point for expression and purification of the uniformly ^15^N, ^13^C, ^2^H-labeled protein needed for 3D NMR experiments. We show the first experimental evidence for how the protein traverses the membrane, and how the individual amino acid residues contribute to the secondary structure of the protein. We establish that OEP16 consists of four TM helices and identify the residues for helix formation. Relaxation measurements report on intramolecular dynamics [11], [12], and help to estimate the isotropic global correlation time that provides insight into the multimeric state of the protein, which is under debate in the literature. Chemical shift perturbation is used to elucidate the residues that are responsible for the selectivity and diffusion of amino acid molecules through OEP16. Results of this study reveal nearly complete assignment of the N, H, CO, C_α_, and C_β_ chemical shifts, which is essential for a NMR high-resolution structure. In addition, a functional study performed via arginine titration provides data that reveals specific ligand binding to OEP16.

## Materials and Methods

### Protein Expression and Purification

For the NMR experiments, U-^13^C,^15^N-labeled and 80% perdeuterated recombinant OEP16 was expressed by using a slightly modified procedure compared to Ni et al. [6]. A 5-mL preculture was prepared in Lysogeny Broth (LB) media that was allowed to incubate at 310 K overnight, shaking at 200 rpm. The preculture was added directly to a 1-L culture of M9 media, with ^15^N-ammonium chloride as the sole ^15^N-nitrogen source and ^13^C-glucose as the sole ^13^C-carbon source. Perdeuteration was achieved by growing the cells in 80% ^2^H_2_O solution. For experiments only requiring ^15^N-labeled protein, cell growth was performed in a similar manner without perdeuteration, employing ^15^N-ammonium chloride as the sole nitrogen source, and non-enriched D-glucose as the carbon source.

Protein expression was induced at OD_600_ = 0.8 using 1 mM isopropyl-β-D-1-thiogalactopyranoside (IPTG) [6], and allowing it to incubate for an additional 5 h. Purification and reconstitution of OEP16 in SDS micelles was carried out using previously developed methods [6]. Protein was prepared for the NMR experiments by diluting the concentrated samples in a 10% ^2^H_2_O buffer (100 mM NaCl, 20 mM NaH_2_PO_4_ pH 6.5, 1 mM β-mercaptoethanol (BME), 1 mM ethylenediaminetetraacetic acid (EDTA), 10% (v/v) glycerol, 0.4% (w/v) SDS, and 0.02% (w/v) NaN_3_). Samples were then re-concentrated via ultrafiltration using a 30 kDa molar mass cut-off (MWCO) filter centricon. U-^15^N labeled OEP16 in β-D-dodecylmaltopyranoside (β-DDM) was prepared in a similar manner, replacing SDS as the detergent used to form OEP16 micelles.

### Protein Backbone Assignments

NMR data were collected at 310 K (with the exception of one ^1^H-^15^N HSQC spectrum collected at 298 K) using an INOVA 600 Varian NMR spectrometer equipped with a triple-resonance cryoprobe. Sample condition screening and chemical shift assignments were carried out by collecting a series of 2D and 3D NMR experiments. The ^15^N-HSQC, HNCA, HN(CO)CA, HNCACB, CBCA(CO)NH, HNCO, HN(CA)CO, TOCSY-^15^N-HSQC, and NOESY-^15^N-HSQC experiments were performed by standard pulse sequences [13]–[20]. The NOESY and TOCSY mixing times were 80 ms and 50 ms respectively. Additional NMR data for OEP16 in β-DDM micelles were acquired at 318 K with a Varian NMR System (VNMRS) operating at 800 MHz using a ^1^H{^13^C/^15^N} 5 mm XYZ PFG triple-resonance room temperature probe. Data were processed with NMRPipe [21], while SPARKY [22] was used for resonance assignments performed by sequentially walking through the backbone, using C_β_ and C_α_ chemical shift statistics from the Biological Magnetic Resonance Data Bank (BMRB) for identification of amino acid residues [23]. Assigned chemical shifts were analyzed for secondary structure via torsion angles using TALOS+ [24], [25].

### Relaxation Measurements

The ^15^N longitudinal Zeeman (*T*_1*Z*_) and transverse (*T*_2_) relaxation data were acquired on the singly labeled (^15^N) protein sample for relaxation analysis using standard experiments [26]. Relaxation delay times ranging from 10 ms to 2000 ms were used for *T*_1*Z*_ data, while delay times between 10 ms and 190 ms were used for obtaining the *T*_2_ data. Data were processed using NMRPipe [21]. Initial relaxation analysis for calculating *T*_1*Z*_ and *T*_2_ values was performed using SPARKY [22]. The average overall correlation time (*τ*_m_) of the protein was estimated from the relaxation data in the limit of highly restricted internal motions [27]–[29].

### Amino Acid Titration

For observing the amino acid residues of OEP16 involved with substrate binding, 500 mM arginine in a 10% ^2^H_2_O buffer (100 mM NaCl, 50 mM NaH_2_PO_4_ pH 7.4, 1 mM BME, 1 mM EDTA, 10% (v/v) glycerol, 0.4% (w/v) SDS, and 0.02% (w/v) NaN_3_) was titrated directly into the NMR tube containing 1.2 mM OEP16 and 100 mM NaCl, 20 mM NaH_2_PO_4_, pH 7.4, 1 mM BME, 1 mM EDTA, 10% (v/v) glycerol, 0.4% (w/v) SDS, and 0.02% NaN_3_. Arginine concentrations of 0 mM, 10 mM, 20 mM, and 40 mM, were used for the titration studies of the singly-labeled ^15^N protein sample. The ^1^H and ^15^N shifts chemical shifts were obtained using a 2D ^15^N-HSQC experiment.

## Results

### Backbone Residue Assignments

**Figure 1:**
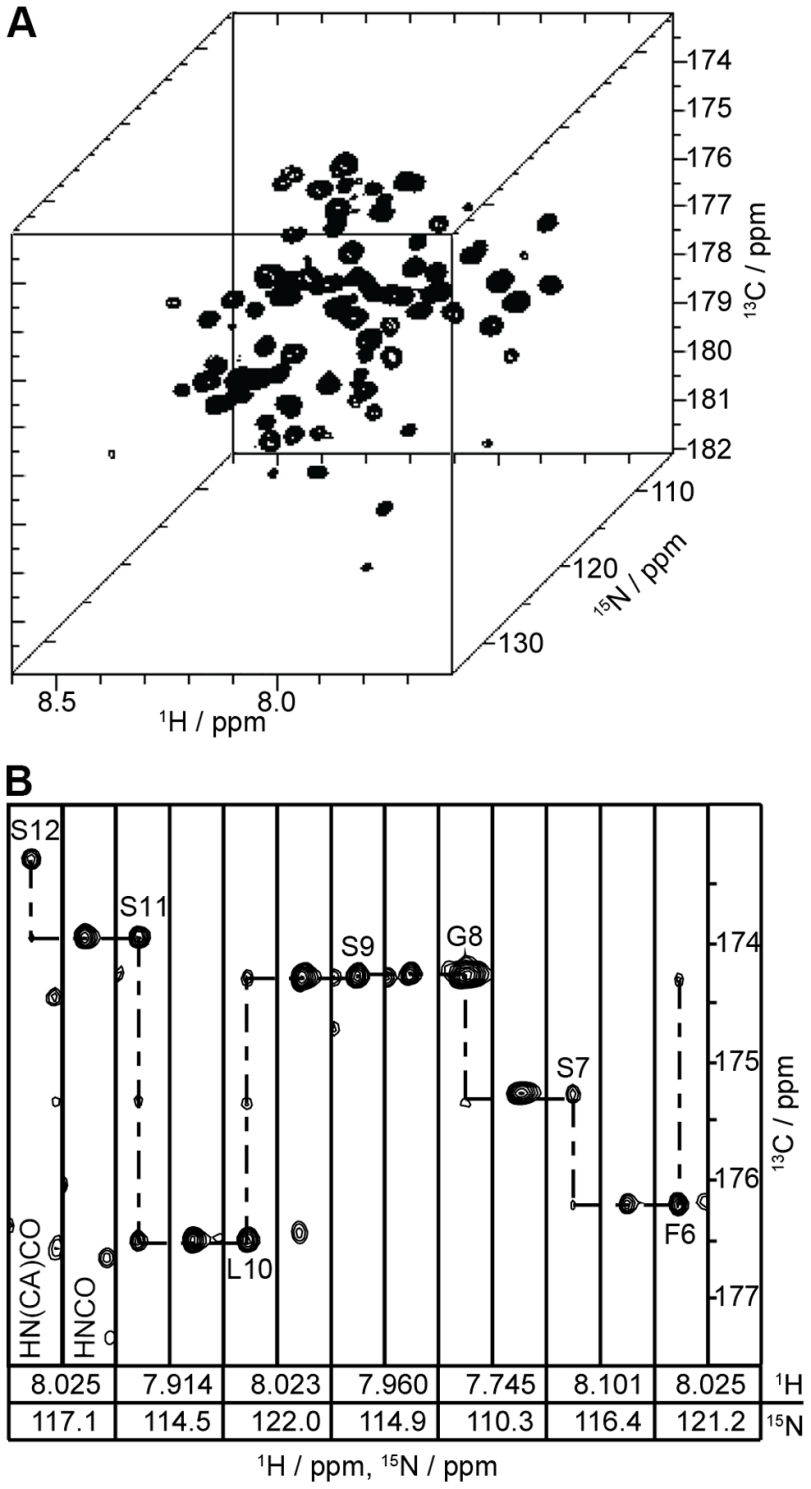
Three-dimensional NMR spectroscopy of U-^15^N,^13^C-labled OEP16 in SDS micelles provides sequential backbone assignments. (A) The HNCO experiment result in well-resolved peaks that aided in the assignment of over 95% of the ^15^N and ^13^C resonances. (B) Section of a strip plot from the HN(CA)CO and HNCO spectra. Spectra were recorded for OEP16 at 600 MHz in 0.4% SDS micelles, containing 20 mM NaH_2_PO_4_ buffer pH 6.5 and 100 mM NaCl at 310 K.

**Figure 2:**
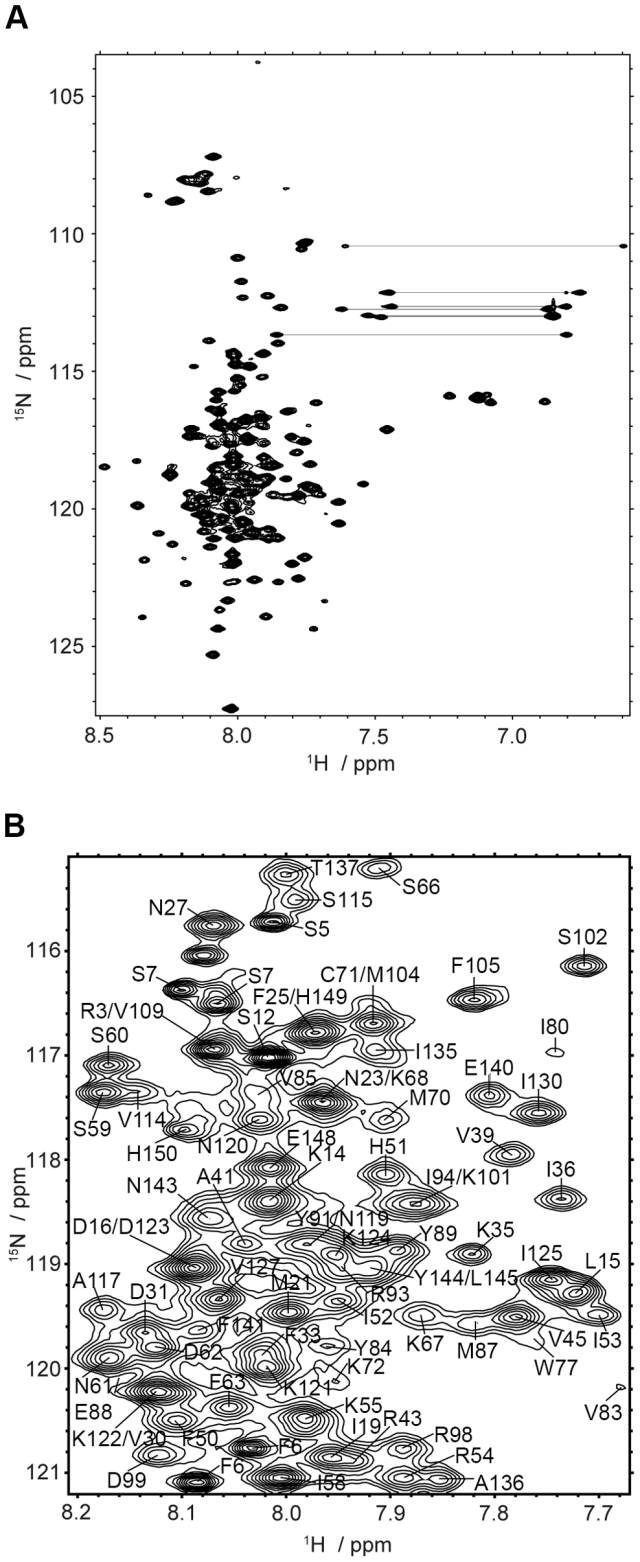
Two-dimensional ^1^H-^15^N HSQC spectra of OEP16 in SDS detergent micelles show well-dispersed peak patterns indicative of secondary structure. (A) ^1^H-^15^N-HSQC spectrum obtained at 600 MHz for OEP16 in SDS detergent micelles at 310 K. Sample conditions are identical to Figure 1. Horizontal lines connect the amino side chain resonances. (B) Expansion of the central crowded region of ^1^H-^15^N HSQC spectrum of OEP16, showing the polypeptide backbone resonance peak assignments corresponding to the amino acid sequence. Assignable resonance peak patterns are observed despite the narrow ^1^H chemical shift dispersion.

Very good yields of purified OEP16 in SDS micelles were obtained for the singly U-^15^N-labeled and U-^13^C, ^15^N, ^2^H-labeled samples. The 80% perdeuterated ^13^C, ^15^N sample preparations on average involved a 600-µL solution that contained 1 mM OEP16. Typical singly-labeled ^15^N sample preparations entailed a 1-mL solution at a protein concentration of 1.5 mM. The relatively high protein concentrations provided very good signal-to-noise ratios for all NMR experiments described in the study as indicated in Figure 1A. We were able to assign 95% of the carbon, nitrogen, and proton backbone resonances with the experiments described. Fairly well-resolved spectra were seen in all experiments conducted at 600 MHz. Figure 1B shows a segment of a strip plot using HNCO and HN(CA)CO chemical shifts to sequentially assign the backbone of OEP16. Only residue M1 and the C_β_ shift for Y144 could not be confidently assigned. A representative 2D ^15^N-^1^H HSQC spectra is shown in Figure 2A, which includes non-backbone amide resonances due to arginine, asparagine, glutamine, tryptophan, and, lysine side chains. The assigned resonances are shown in the expansion of the 2D ^15^N-HSQC spectrum in Figure 2B and a complete list of assigned resonances can be accessed via the Biological Magnetic Resonance Bank (BMRB), accession number 19267.

Among the amino acid assignments, seven residues (F6, S7, G8, S44, L67, G74, and A75) as shown in Figure S1 were found to have two peaks in the 2D ^15^N HSQC spectrum, but maintained the same CO, C_α_, C_β_, and H_α_ chemical shifts, which may suggest two isoforms. This interesting finding is further addressed in the discussion. The combined HN(CA)CO, HNCO, HNCA, HN(CO)CA, HNCACB, and CBCA(CO)NH data provided the necessary information to assign nearly 95% of the protein using strip plot methods to sequentially walk through the backbone. Short range NOEs between the H_α_ and the amide protons provided by the NOESY-HSQC experiment clarified resonance assignments that were ambiguous in the ^13^C NMR spectra. A complete list of the assigned resonances is provided in Table S1.

### Helix Prediction

**Figure 3:**
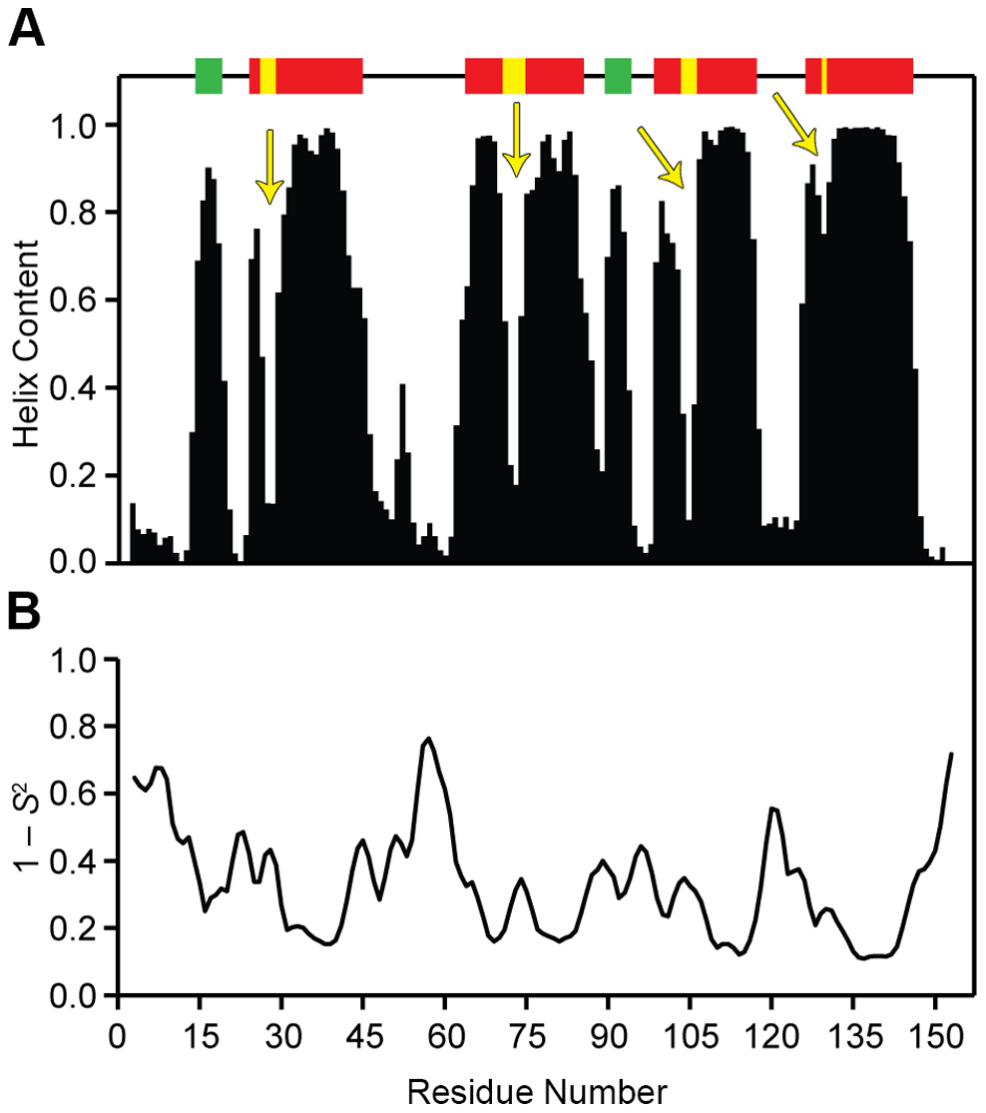
Analysis of backbone chemical shifts of OEP16 in SDS detergent micelles using TALOS+ program. (A) Predictions of the α-helical structure of OEP16 and (B) estimated ^1^H-^15^N bond orientational order parameters (1-*S* ^2^) as a function of residue position in protein amino acid sequence. The suggested TM regions are indicated by the red bars above the plot, with yellow arrows indicating the breaks or kinks in the helix. Additionally, the green bars represent the solvent-exposed helices.

Predictions of secondary structure estimated with the program TALOS+ revealed that OEP16 consists of α-helices and loops as seen in Figure 3A. In combination with hydropathy analysis [9] the data suggest the presence of four transmembrane helices with two small extrinsic helical regions. Additionally, TALOS+ evidence suggests that each transmembrane helix displays a break or a possible kink at the N-terminal side of the helix. TALOS+ can also estimate the squared order parameters (*S^2^*) for each residue using the random coil index (RCI) as described [30]. The value of *S^2^* characterizes intramolecular motion in the molecular reference frame [11], [31], [32], which can provide insight into the flexible loop regions of the protein seen in Figure 3B [5]. Flexible regions of the protein are apparent at either end of the polypeptide chain, as well as a large region between residues F49 and S66. The data are used to generate a possible model for OEP16. This model includes areas of the TM helices where there are bends or breaks, as well as two small helices on the surface of the membrane protein.

High-resolution structural restraints are necessary to confirm the presence of a well-folded protein in detergent micelles. However, SDS is not traditionally considered a detergent used for folding proteins. Therefore, a 2D ^15^N-HSQC spectrum was obtained for ^15^N-labeled OEP16 purified in β-D-dodecyl maltopyranoside (β-DDM), a detergent more widely used for structural studies of membrane proteins, at 318 K using an 800 MHz spectrometer. Figure S2 shows the superimposed ^1^H-^15^N-HSQC spectra recorded for OPE16 in SDS micelles versus β-DDM micelles. The overlay of spectra shows a similar resonance peak pattern which strongly suggests that OEP16 is similarly folded in both SDS and β-DDM micelles. Broader peak widths are observed in the β-DDM spectra despite being acquired at a greater field strength for two reasons: the first is that the 600 MHz spectra were acquired to higher resolution (1024 *t*_1_ points compared to the 256 *t*_1_ points for the 800 MHz spectra); additionally the micelle size of β-DDM is nearly four times larger than SDS micelles, which contributes to a significantly longer rotational correlation time (estimated to be ∼26 ns in β-DDM as compared to the calculated 12.5±0.5 ns in SDS as calculated by relaxation analysis), and thus broader resonance peaks despite the greater field strength. Therefore a ^1^H-^15^N HSQC spectrum of OEP16 in SDS acquired at a lower temperature of 298 K is used in the overlay shown in Figure S2. Nonetheless it is possible to identify several of the resolved peaks and assign them based on the assignments from the SDS measurements shown in Figure S2. This provides further evidence of a similar fold of OEP16 in both SDS as well as β-DDM detergent micelles.

### Relaxation Measurements

**Figure 4:**
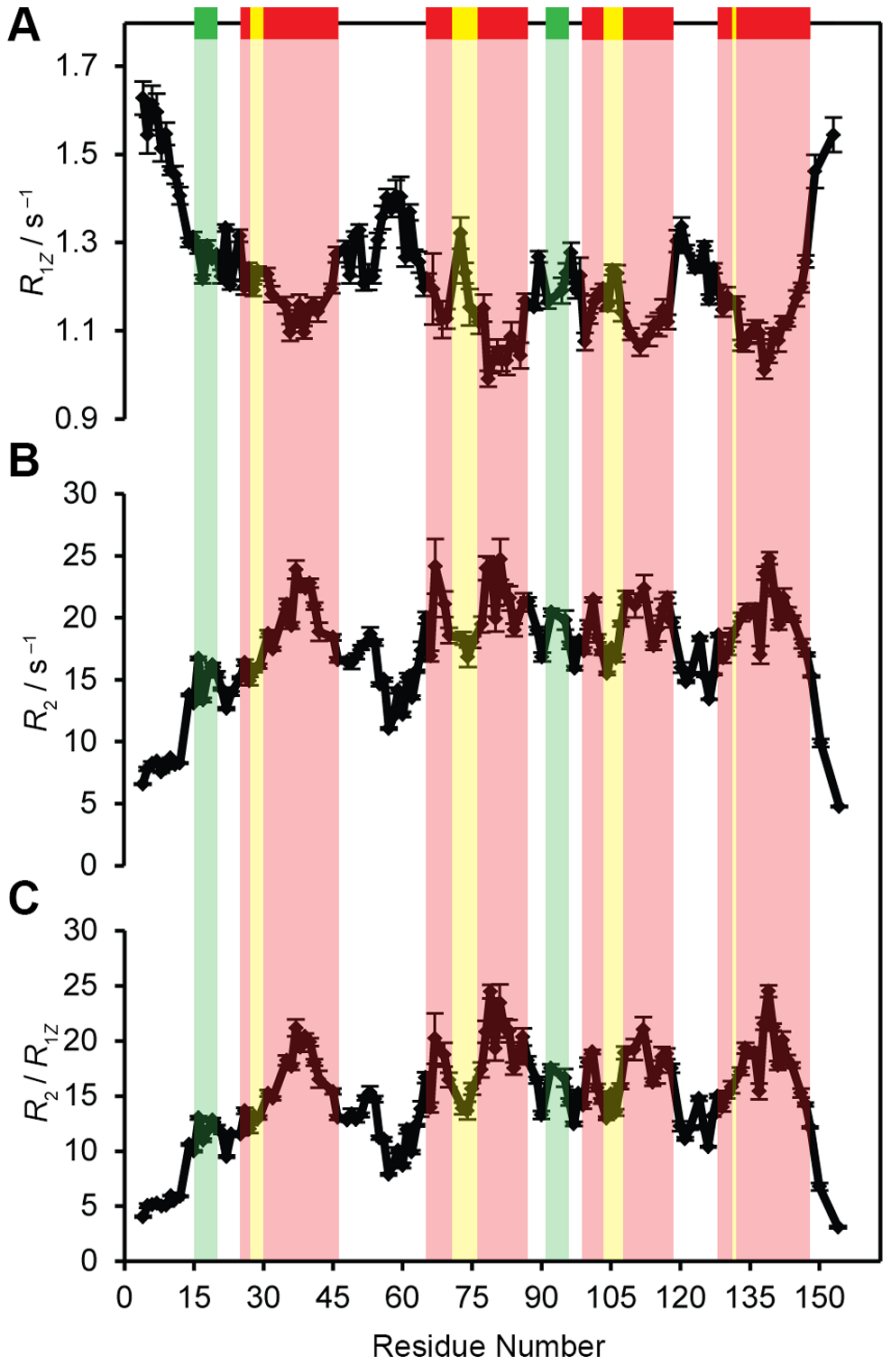
Variations in ^15^N relaxation rates *R*_1Z_ and *R*_2_ and the ratios *R*_2_/*R*_1Z_ support the predicted secondary structure of OEP16 and backbone assignments. (A) ^15^N spin-lattice *R*_1Z_ relaxation rates, (B) ^15^N transverse *R*_2_ relaxation rates, and (C) *R*_2_/*R*_1Z_ ratios. The suggested TM helical regions are indicated by the red bars above the plot with helical breaks in yellow and solvent-exposed helices in green. Relaxation rates for U-^15^N-labeled OEP16 in SDS micelles were obtained at 600 MHz at 310 K with sample conditions identical to Figure 1. Higher *R*_2_ */R*_1Z_ values are located in the central part of the TM helices possibly indicating relatively rigid regions of the protein.

The^ 15^N spin-lattice (*T*_1*Z*_) and spin-spin (*T*_2_) relaxation time values were measured for assigned resonances in the 2D ^15^N-HSQC spectra, and are expressed in terms of the corresponding relaxation rates *R*_1*Z*_ = 1/*T*_1*Z*_ and *R*_2_ = 1/*T*_2_. The ^15^N *R*_1*Z*_ and *R*_2_ values are plotted against residue number in Figure 4A and 4B respectively. They demonstrate a pattern of rising and falling *R*_2_/*R*_1*Z*_ values that are related to the protein backbone and local dynamics of the residues within the protein. Variations of the *R*_2_/*R*_1*Z*_ profile shown in Figure 4C support the predicted secondary structure. Higher flexibility is seen in the loop regions and N- and C-terminal ends of the protein. Assuming the relaxation is due to ^1^H and ^15^N dipolar interactions together with ^15^N chemical shift anisotropy (CSA) modulated by local fluctuations and overall protein tumbling motion, the relaxation rates are given by the following expressions [27], [32], [33]:
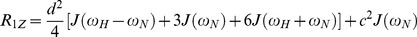
(1)

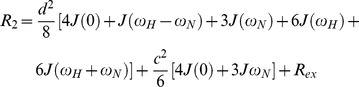
(2)


Here 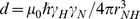, 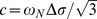, *µ*_0_ is the permeability of free space, *h = *2*πħ* is Planck’s constant, *γ*_H_ and *γ*_N_ are the magnetogyric ratios of the ^1^H and ^15^N nuclei respectively, *r*_NH_ is the ^15^N–^1^H bond length, *ω*_H_ and *ω*_N_ are the Larmor frequencies of the ^1^H and ^15^N spins, and Δσ is the chemical shift anisotropy of the ^15^N spin. Typically peptide bonds demonstrate an axially symmetric chemical shift tensor, and have an average CSA of –160 ppm [28]. Consequently a uniform value of Δσ = –160 ppm was used in this study.

In Eqs. 1 and 2 the spectral densities J(*ω*) are given by [5], [11], [31], [32], [34]:
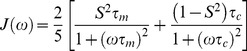
(3)where *S* ^2^ is the generalized ^1^H-^15^N bond orientational order parameter, and *τ_m_* is the isotropic rotational correlation time of the protein molecule. If τ_f_ is the correlation time for internal fast motions, then the effective correlation time can be defined as:



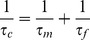
(4)Assuming that internal motions are restricted in their amplitude and fast enough that their contributions can be neglected in the relaxation processes, then J(*ω*) takes the simple canonical form [11], [31] :
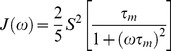
(5)


The expression for *R*_2_/*R*_1*Z*_ is given by:
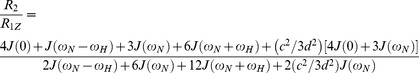
(6)


Note that the *R*_2_/*R*_1*Z*_ ratio is independent of *S* ^2^. Hence one can estimate the local effective correlation time, and thereby anisotropic diffusion tensor, by fitting the experimental *R*_2_/*R*_1*Z*_ data to theoretical expressions given by above equations, assuming the ^15^N-H bond vector orientation distribution is known. However, for a spherical protein, due to lack of a unique principal projection axis, a single correlation time (*τ_m_* = 1/6*D*_iso_) can be defined as the mean value of the effective correlation times independent of N-H bond vector orientation [28], [29]. For determining *τ_m,_* the contributions from residues that are highly mobile and residues responsible for chemical exchange have to be eliminated, so that only residues rigidly bound to protein are used.

Accordingly, we calculated the local effective correlation times by fitting the experimental *R*_2_/*R*_1*Z*_ values for each residue to Eq. 6. In our calculations, because we do not have the N-H orientation data, as a first approximation we estimated the isotropic overall correlation time by assuming OEP16 to be a nearly spherical protein. In this process, we did not consider the highly mobile residues (first 10 residues from N-terminal end and last two residues from C-terminal end). To eliminate the residues responsible for chemical exchange, we chose the residues with *R*_2_ values higher than one standard deviation from the average *R*_2_ value. In fact none of the residues show such high *R*_2_ values. Here we considered all other residues to estimate *τ_m_*. The loop region (residue numbers 55–65) is relatively flexible but inclusion of those residues did not show much impact in the average correlation time calculations.

The assumption of a single isotropic rotational correlation time is a simplification for most macromolecules. However, the anisotropic effects are small for slightly non-spherical proteins. Here, the calculated *τ_m_* value is used as a qualitative signature of the presence of a monomeric OEP16 molecule. It is not intended to explain the anisotropic diffusion parameters, for which a 3D structure either from X-ray crystallography or NMR spectroscopy would be necessary. That is beyond the scope of the present study, and is not needed to substantiate our major findings. The estimated *τ_m_* value is typically taken as the initial value for the model-free analysis. The assumption is that if a dimer or multimer state exists, it would be reflected in the average overall tumbling time corresponding to slowing down of the motions. Previously, such an estimation of effective correlation times was established in the case of a 18 kDa protein [28], and those correlation times were used to estimate the order parameters. Furthermore, we expect the variations observed in *R*_2_/*R*_1Z_ ratio are predominantly the signatures of dynamics of the α-helices, rather than the internal motions or exchange. Notably the plots of *R*_2_ and R_2_/*R*_1*Z*_ as a function of residue number reflect a similar trend as the TALOS+ predicted secondary structure of OEP16. We observe higher *R*_2_ values and hence *R*_2_/*R*_1*Z*_ values in the middle of helices than the edges and loops. Such a trend in relaxation rates reflects the secondary structure on the one hand, and supports the backbone assignments on the other.

The average overall global correlation time of 12.5±0.5 ns can then be used to estimate a hydrodynamic radius of 2.4 nm by applying Stoke’s Law [5]:
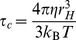
(7)


In this expression *η* is the viscosity of the solvent, estimated to be 0.931 cP for a 10% glycerol-water solution at 310 K [35], *r_H_* is the hydrodynamic radius of the protein-micelle complex, *k*_B_ is the Boltzmann constant, and *T* is temperature. Additionally, the molar mass of the complex can be estimated from *r_H_* [5] if the protein density is assumed to be ρ = 1.37 g/cm^3^ (and therefore a protein-micelle density of ρ = 1.18 g/cm^3^) and the hydration layer is estimated to be 1.6 Å (corresponding to one-half a hydration shell):
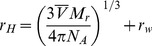
(8)


Here *N_A_* is Avogadro’s number, and 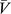 is the specific volume of the protein. For OEP16 a molar mass (*M_r_*) of 31.8 kDa is calculated. This value corresponds to an OEP16 monomer plus 65 SDS molecules forming the micelle. An estimated correlation time of an OEP16 dimer with a sufficient SDS micelle would correspond to between 18–19 ns which is significantly larger than what was measured. Although the assumptions necessary for the molar mass calculation prevent an exact number, the conclusion of a monomeric protein can be made with confidence.

### Arginine Titration Results

**Figure 5:**
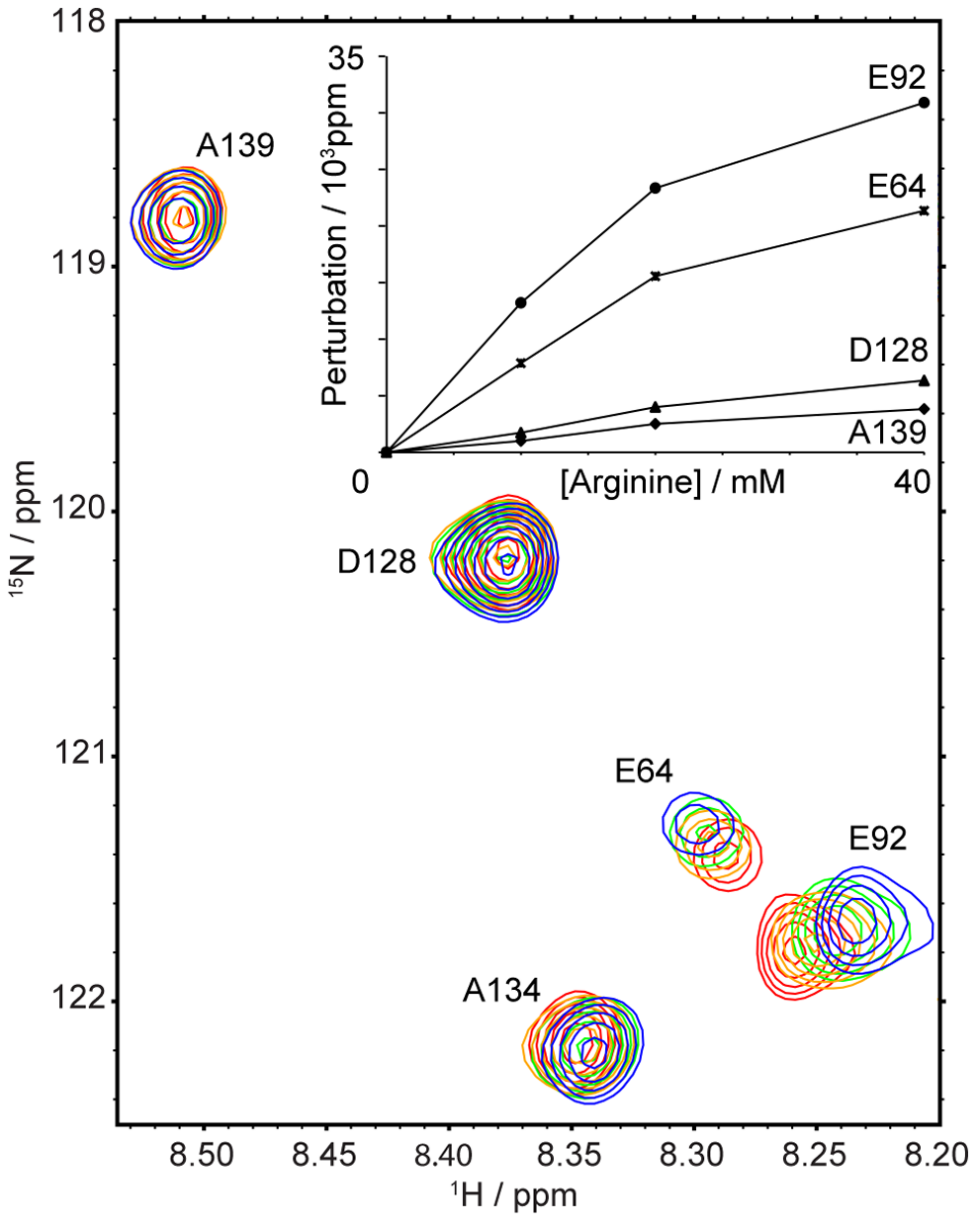
Arginine titration studies reveal the ligand binding regions of OEP16. Chemical shift perturbations for the most significantly affected residues of wild-type OEP16 are plotted as function of arginine concentration (shown in inset). A 500-mM arginine solution in a 10% ^2^H_2_O buffer containing 100 mM NaCl, 50 mM NaH_2_PO_4_ pH 7.4, 1 mM BME, 1 mM EDTA, 10% (v/v) glycerol, 0.4% (w/v) SDS, and 0.02% (w/v) NaN_3_ was titrated into the NMR tube for final concentration values of 0 mM, 10 mM, 20 mM, and 40 mM of arginine. The hyperbolic shape of the binding curves suggests the specific binding of arginine to OEP16. Nonspecific binding residues D128 and A139 show a distinctively linear relationship upon arginine titration. Blue indicates the spectrum in the absence of arginine and progresses to green, then orange, and ultimately red as increasing amounts of arginine are added.

A number of significant chemical shift perturbations were observed throughout the protein upon titration with increasing amounts of arginine as depicted in Figure 5. Specific binding yields a rectangular hyperbola with a saturating end point similar to what is shown for E64 and E92 (the two most prominent shift perturbations) in the inset of Figure 5. The *y*-axis reports the scaled chemical shift perturbation across both ^15^N and ^1^H dimensions (scaled according to Ref. [36]). The chemical shift perturbation via the introduction of arginine displays specific, weak binding of the amino acid to the OEP16 monomer. The residues show significant chemical shift perturbations which are nonlinear compared to the other residues, such as D128 and A139, which suggests that any binding that occurs at these sites is strictly nonspecific.

### OEP16 Topology

Taken together, the TALOS+ calculations and relaxation data provide information on how the protein crosses the membrane when the hydrophobicity analysis [9] also considered. The first TM helix begins at residue F25 and spans the membrane to S44; the second TM helix starts at residue E64 and traverses the membrane ending at residue Y89; the peptide crosses the membrane a third time at residue N102 and ends at residue N119; and finally the fourth TM region begins at residue V127 and ends at residue T146, as illustrated in Figure S3. Figure S3 demonstrates that the independent calculations and measurements align well with each other along with the hydropathy data generated from the protein’s primary structure. The α-helix prediction by TALOS+ also suggests that there is a small break within each of the four TM helical regions, at N27, K72, A103, and I130. Order parameters and relaxation data suggest that these residues are flexible relative to the surrounding residues in the sequence.

## Discussion

In this work we have addressed three important properties of the membrane protein OEP16: the formation of transmembrane helices of OEP16 protein, its likely monomeric state in SDS detergent micelles, and the ligand binding properties of this protein using results obtained from various high-resolution NMR experiments. The three-dimensional NMR data for the OEP16 protein have been used to obtain 95% of the amino acid backbone chemical shift assignments. These chemical shift data have been analyzed using the TALOS+ program to detect the secondary structure elements, and to estimate ^1^H-^15^N bond orientational order parameters. The evaluated secondary structure reveals that OEP16 contains four α-helices connected by flexible loop regions. The overall α-helix content calculated in this method (∼55%) fits well with previously measured circular dichroism [6] and hydropathy plot data (∼50%) [9].

**Figure 6:**
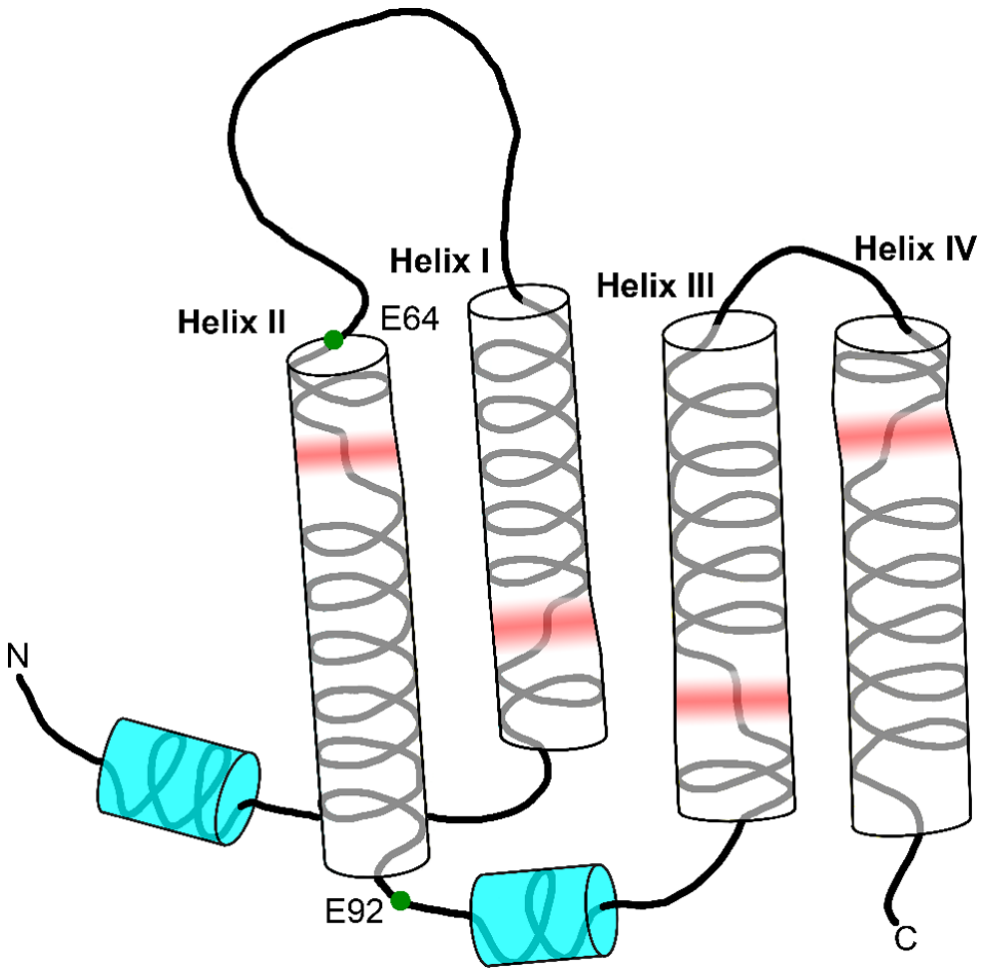
illustration of secondary fold based on evidence in this study. Side view of the protein is shown to demonstrate how the helices traverse the membrane, and indicates amino acids involved with ligand transport. Included in red are the locations where there are possible breaks or kinks in the helices.

Notably, the information unveiled in these studies provides the first experimental evidence that OEP16 consists of four TM helices, and identifies the residues involved with helix formation. The presence of four α-helical based on our results coincides with the four-helix model inferred from residue hydropathy analysis [9]. Moreover, the amount of helix content calculated by TALOS+ is in agreement with the CD data provided in previous studies [6], [9]. By comparing TALOS+ predicted α-helices regions and ^1^H-^15^N bond orientational order parameters with the ^15^N relaxation data, one can locate the TM helices as shown in Figures 3 and 4. The breaks in the TM α-helices provide additional structural information. The location of each of these breaks is interesting as well: they all begin at the N-terminal part of the helix as it traverses the membrane. This means that when the N-terminus of the protein is oriented “down” in the membrane, the breaks for H1 and H3 are closer to the bottom, while the breaks for H2 and H4 helices are located at the top. 6 All of the experimental results and conclusions are pictorially summarized in Figure 6. It is possible that these flexible regions function either for specificity of substrate diffusion, or play a role in opening and closing the channel as a way for the amino acids to diffuse into the chloroplast. Interestingly, the seven identified residues with two peaks in the ^15^N-HSQC may suggest that OEP16 may exist as two different conformations in slow exchange on the NMR time scale. This may provide a mechanistic insight to OEP16 when a high-resolution structure is obtained. As an example it is possible that one isoform is in a conformation that inhibits amino acid diffusion across the outer chloroplast membrane versus the other conformation.

The chemical shift perturbation study agrees with mutation studies, which show that H1 and H2 are required for protein function. Chemical shift data describes regions of the protein where α-helices are present, and relaxation results point to the flexible loop regions of the protein. Due to the selectivity of OEP16 for amino acids, the arginine titration experiment identifies residues within OEP16 where the ligand directly interacts. This study provides insight into the mechanism of OEP16 function, although more indepth studies are required to provide further detailed information. An additional aspect addressed in this study is the possibility of forming oligomeric states of OEP16. The multimeric state of OEP16 has been a topic of discussion in the past literature, with dimer formation imposed from cross-linking cysteine residues, [7] whereas trimers are suggested from electron micrograph data [10]. Moreover, previous gel filtration results have suggested a larger multimeric state [6]. Nonetheless the average rotational correlation time of ∼12.5 ns calculated by the relaxation data clearly suggests the presence of an OEP16 monomer in SDS micelles. Although these calculations assume OEP16 as a rigid globular protein, for the overall tumbling motion of simple rigid proteins of the size of 16 kDa, these time scales can be well established. Hence one can conclude that OEP16 predominantly forms monomers in SDS micelles.

The data presented in this study represents the penultimate step in understanding the functional and structural characteristics of OEP16. The information obtained will be crucial for a three-dimensional structure. Future experiments will involve amino acid specific labeling in order to take advantage of the structural information provided by long range NOEs. Orienting OEP16 in a weakly aligning media such as polyacrylamide will also provide structural refinement via residual dipolar couplings.

## Supporting Information

Figure S1 Two-dimensional ^1^H-^15^N HSQC spectra indicate the possibility of conformational exchange of OEP16 solubilized in SDS detergent micelles. The spectrum was acquired at 600 MHz and sample conditions are identical to Figure 1. Seven residues have two peaks each in the 2D ^15^N-HSQC spectrum and are shown in the boxes: (A) F6, (B) S7, (C) G8, (D) S44, (E) L67, (F) G74, and (G) A77 all have two peaks, but have identical ^13^C_α_, ^13^C_β_, ^13^CO, and ^1^H_α_ chemical shifts; (H) control showing single peaks. The different chemically shifted resonances may indicate two different conformations of the protein in slow exchange on the NMR time scale.
(TIF)

Figure S2 A comparison of ^1^H-^15^N HSQC spectra of OEP16 in SDS micelles and β-DDM micelles**.** Similarities in protein secondary structure and folding are clearly evident. The spectrum of OEP16 in SDS micelles (red) obtained at 600 MHz at 298 K is superimposed onto the spectrum of the protein in β-DDM (blue) obtained at 800 MHz. at 318 K. Inset is a ^1^H-^15^N HSQC spectrum of OEP16 in SDS at 310 K (black) superimposed on a spectrum of OEP16 in SDS at 298K (red), both obtained at 600 MHz. Several resolved resonances are assigned. Sample conditions are identical to Figure 1. The similar peak positions of the backbone amides suggest that OEP16 folds similarly in the nonionic detergent β-DDM and the anionic detergent SDS. Linewidths are significantly broader at 298K compared to 310K, and are very similar to the linewidths of OEP16 in β-DDM despite the temperature at which the spectra were acquired.
(TIF)

Figure S3 Transmembrane regions of OEP16 are predicted using different methods and are shown together for comparison. Results of relaxation measurements (red), TALOS+-predicted secondary structure (green), and *S* ^2^ values (purple) are indicated. Orange represents the comparisons to predictions of previously published hydropathy plot analysis [7]. Beginning and ending residues differ slightly yet the data are in agreement for the general location of the TM helices.
(TIF)

Table S1 Assigned chemical shifts for each residue in OEP16. Assignments were made using a combination of HNCO, HN(CA)CO, HNCA, HN(CO)CA, HNCACB, CBCA(CO)NH, ^15^N-TOCSY-HSQC, and ^15^N-NOESY-HSQC experiments.
(DOC)
