# Supplementary figures and images for: High-Resolution NMR Reveals Secondary Structure and Folding of Amino Acid Transporter from Outer Chloroplast Membrane
Source: PLoS One. 2013 Oct 29;8(10):e78116. doi: 10.1371/journal.pone.0078116 (PMC3812221; doi:10.1371/journal.pone.0078116)

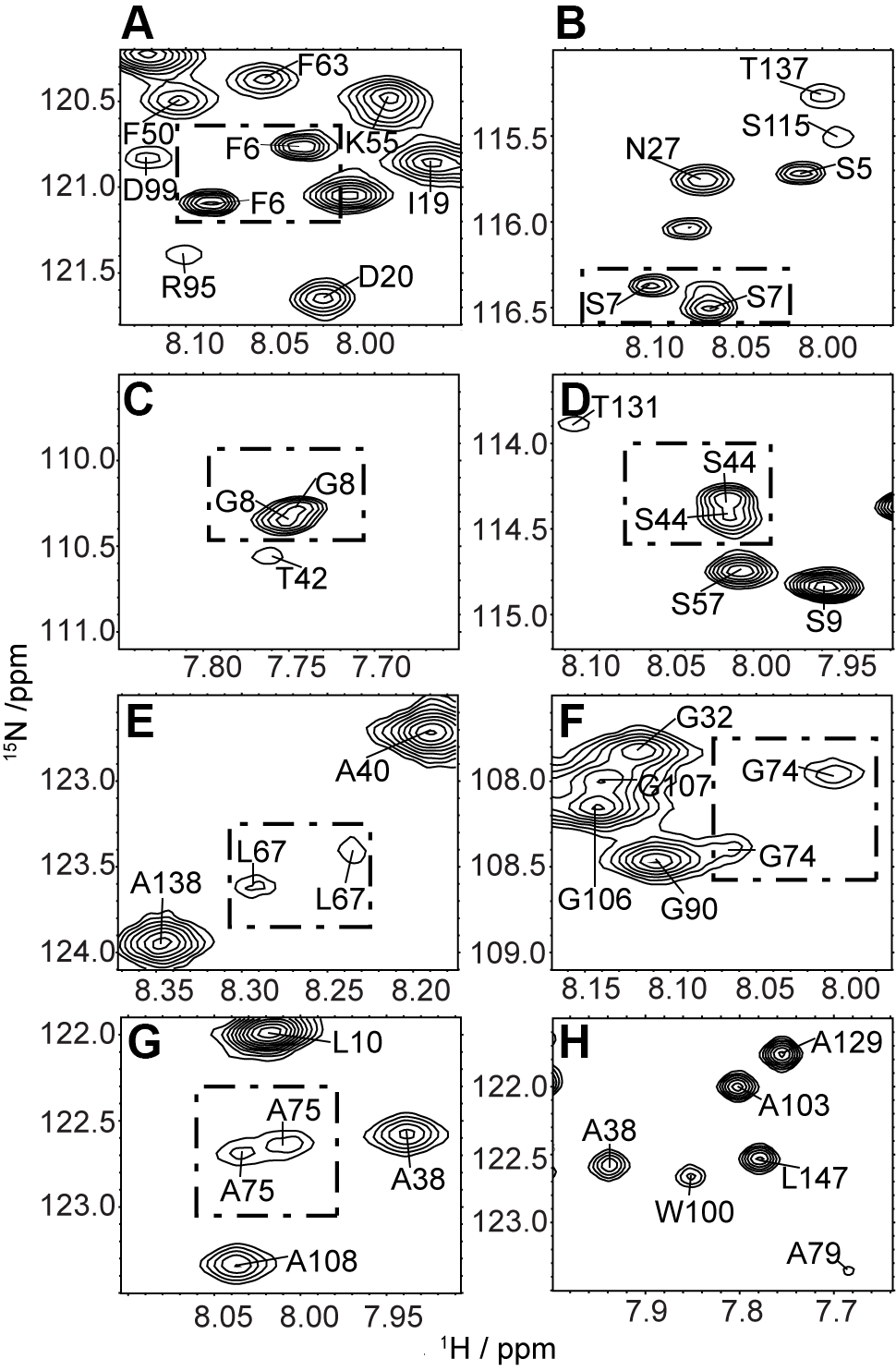

Supplement: Figure S1 — Two-dimensional 1H-15N HSQC spectra indicate the possibility of conformational exchange of OEP16 solubilized in SDS detergent micelles. The spectrum was acquired at 600 MHz and sample conditions are identical to Figure 1. Seven residues have two peaks each in the 2D 15N-HSQC spectrum and are shown in the boxes: (A) F6, (B) S7, (C) G8, (D) S44, (E) L67, (F) G74, and (G) A77 all have two peaks, but have identical 13Cα, 13Cβ, 13CO, and 1Hα chemical shifts; (H) control showing single peaks. The different chemically shifted resonances may indicate two different conformations of the protein in slow exchange on the NMR time scale. (TIF) [file pone.0078116.s001.tif]

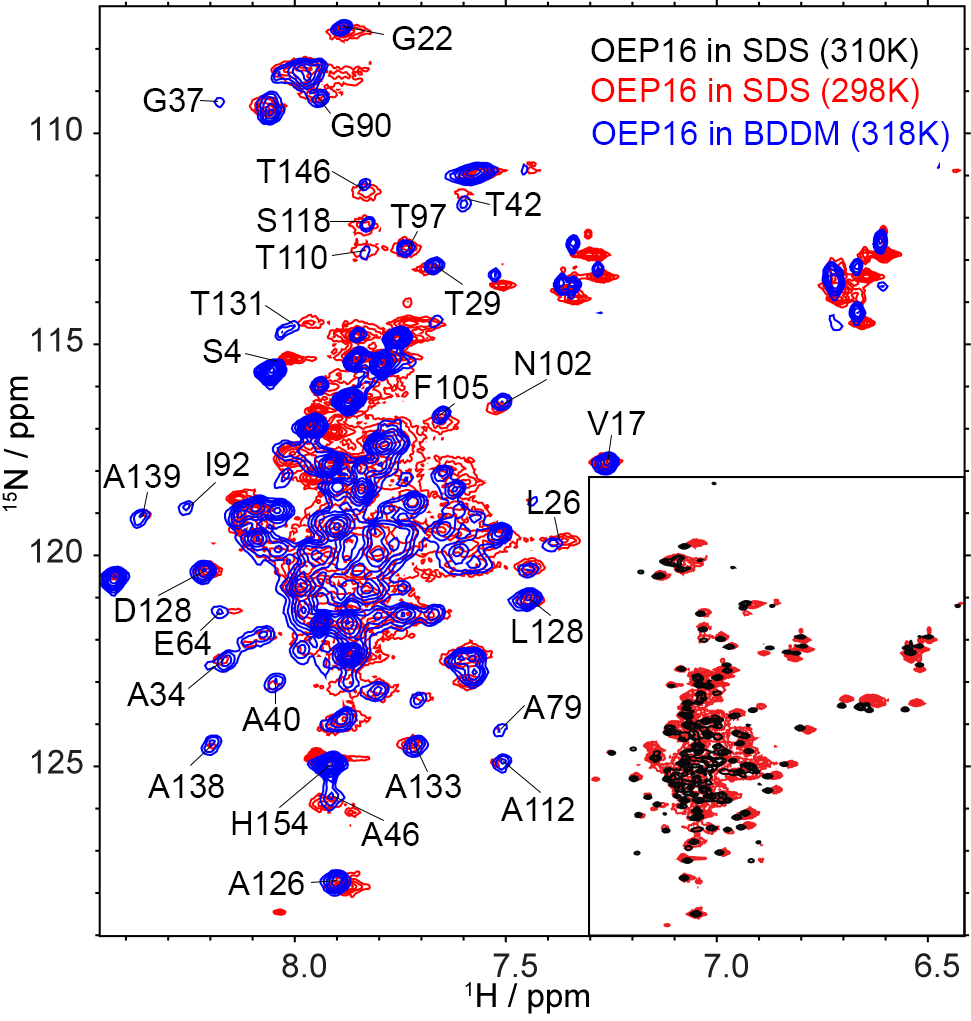

Supplement: Figure S2 — A comparison of 1H-15N HSQC spectra of OEP16 in SDS micelles and β-DDM micelles. Similarities in protein secondary structure and folding are clearly evident. The spectrum of OEP16 in SDS micelles (red) obtained at 600 MHz at 298 K is superimposed onto the spectrum of the protein in β-DDM (blue) obtained at 800 MHz. at 318 K. Inset is a 1H-15N HSQC spectrum of OEP16 in SDS at 310 K (black) superimposed on a spectrum of OEP16 in SDS at 298K (red), both obtained at 600 MHz. Several resolved resonances are assigned. Sample conditions are identical to Figure 1. The similar peak positions of the backbone amides suggest that OEP16 folds similarly in the nonionic detergent β-DDM and the anionic detergent SDS. Linewidths are significantly broader at 298K compared to 310K, and are very similar to the linewidths of OEP16 in β-DDM despite the temperature at which the spectra were acquired. (TIF) [file pone.0078116.s002.tif]

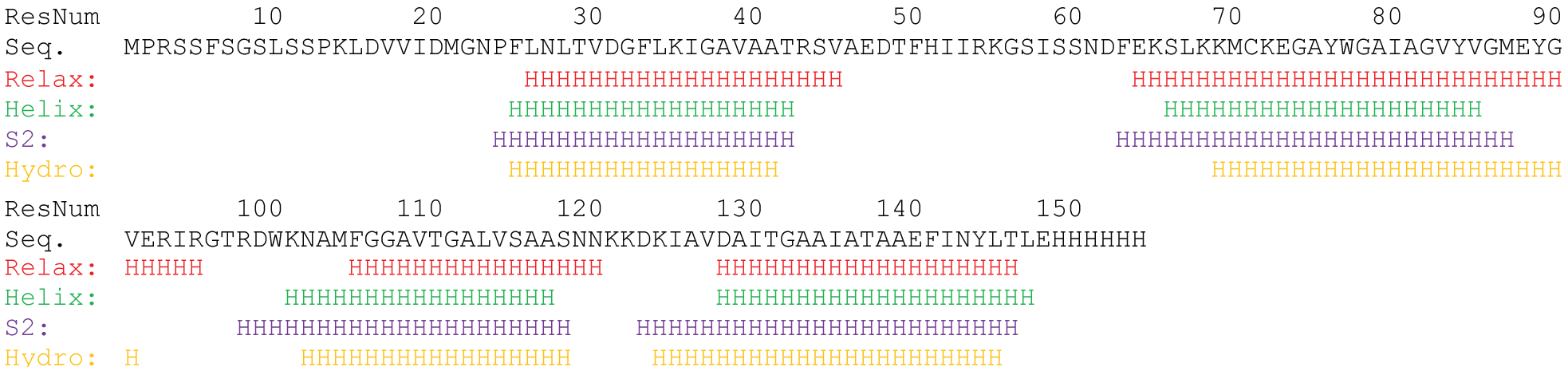

Supplement: Figure S3 — Transmembrane regions of OEP16 are predicted using different methods and are shown together for comparison. Results of relaxation measurements (red), TALOS+-predicted secondary structure (green), and S 2 values (purple) are indicated. Orange represents the comparisons to predictions of previously published hydropathy plot analysis [7]. Beginning and ending residues differ slightly yet the data are in agreement for the general location of the TM helices. (TIF) [file pone.0078116.s003.tif]
