# Supplementary material for: High-Resolution NMR Reveals Secondary Structure and Folding of Amino Acid Transporter from Outer Chloroplast Membrane
Source: PLoS One. 2013 Oct 29;8(10):e78116. doi: 10.1371/journal.pone.0078116 (PMC3812221; doi:10.1371/journal.pone.0078116)
Supplement: Table S1 — Assigned chemical shifts for each residue in OEP16. Assignments were made using a combination of HNCO, HN(CA)CO, HNCA, HN(CO)CA, HNCACB, CBCA(CO)NH, 15N-TOCSY-HSQC, and 15N-NOESY-HSQC experiments. (DOC) [file pone.0078116.s004.doc]

|  | **CO** | **C_α_** | **C_β_** | **H_N_** | **H_α_** | **N** |
| --- | --- | --- | --- | --- | --- | --- |
| **P2** | 176 | 63.87 | 31.48 | - | - | - |
| **R3** | 176.2 | 56.57 | 30.1 | 8.074 | 4.338 | 117 |
| **S4** | 174.4 | 58.42 | 63.88 | 8.164 | 4.439 | 114.9 |
| **S5** | 174.3 | 58.41 | 63.74 | 8.018 | 4.427 | 115.8 |
| **F6** | 176.2 | 58.08 | 39.1 | 8.091 | - | 121.2 |
| **S7** | 175.3 | 58.88 | 63.69 | 8.101 | 4.338 | 116.4 |
| **G8** | 174.2 | 45.42 | - | 7.745 | - | 110.3 |
| **S9** | 174.3 | 58.72 | 64.09 | 7.96 | 4.489 | 114.9 |
| **L10** | 176.5 | 55.08 | 41.48 | 8.023 | 4.38 | 122 |
| **S11** | 173.9 | 57.78 | 63.96 | 7.914 | 4.499 | 114.5 |
| **S12** | 173.3 | 55.99 | 63.86 | 8.025 | 4.753 | 117.1 |
| **P13** | 177.3 | 63.64 | 31.62 | - | - | - |
| **K14** | 176.8 | 56.87 | 32.23 | 8.016 | 4.267 | 118.4 |
| **L15** | 177.2 | 55.7 | 41.59 | 7.722 | 4.215 | 119.3 |
| **D16** | 176.9 | 55.67 | 40.13 | 8.093 | 4.444 | 119.1 |
| **V17** | 176.4 | 63.26 | 32.19 | 7.463 | 4.022 | 117.2 |
| **V18** | 176.5 | 63.51 | 31.98 | 7.639 | 3.999 | 119.8 |
| **I19** | 176.5 | 62 | 37.96 | 7.963 | 3.986 | 120.9 |
| **D20** | 176.7 | 54.62 | 40.93 | 8.026 | 4.615 | 121.7 |
| **M21** | 176.8 | 56.45 | 32.62 | 8.004 | 4.319 | 119.5 |
| **G22** | 173 | 45.03 | - | 8.092 | - | 107.3 |
| **N23** | 174.3 | 51.7 | 38.94 | 7.969 | 4.855 | 117.6 |
| **P24** | 177.3 | 63.91 | 31.31 | - | - | - |
| **F25** | 176.6 | 58.73 | 38.37 | 7.973 | 4.497 | 116.8 |
| **L26** | 177.6 | 56.29 | 41.53 | 7.553 | 4.183 | 119.2 |
| **N27** | 175.2 | 53.79 | 38.91 | 8.071 | 4.7 | 115.8 |
| **L28** | 176.7 | 55.59 | 42.21 | 7.638 | 4.38 | 120.6 |
| **T29** | 175 | 61.38 | 70.45 | 7.844 | 4.502 | 112.7 |
| **V30** | 176.4 | 64.17 | 31.64 | 8.112 | 3.973 | 120.3 |
| **D31** | 177.7 | 55.86 | 40.61 | 8.135 | 4.479 | 119.7 |
| **G32** | 174.9 | 46.29 | - | 8.113 | 3.891 | 107.9 |
| **F33** | 176.9 | 59.91 | 38.92 | 8.028 | 4.399 | 119.9 |
| **L34** | 178.6 | 56.73 | 40.92 | 8.028 | - | 118.5 |
| **K35** | 178 | 57.87 | 31.78 | 7.826 | 4.185 | 119 |
| **I36** | 177.6 | 63.32 | 37.4 | 7.74 | 3.87 | 118.4 |
| **G37** | 175.1 | 46.63 | - | 8.321 | - | 108.5 |
| **A38** | 179.9 | 54.29 | 18.29 | 7.94 | 4.197 | 122.6 |
| **V39** | 177.9 | 65.01 | 31.35 | 7.79 | 3.798 | 118 |
| **A40** | 179.2 | 54.41 | 17.99 | 8.191 | 4.082 | 122.7 |
| **A41** | 179.2 | 53.96 | 18.35 | 8.047 | 4.219 | 118.8 |
| **T42** | 175.6 | 63.51 | 69.3 | 7.773 | 4.191 | 110.6 |
| **R43** | 176.7 | 57 | 29.83 | 7.934 | 4.242 | 120.9 |
| **S44** | 175.1 | 59.2 | 63.59 | 8.016 | 4.46 | 114.5 |
| **V45** | 176.5 | 62.71 | 31.8 | 7.783 | 4.123 | 119.6 |
| **A46** | 178 | 52.9 | 18.8 | 8.084 | 4.254 | 125.3 |
| **E47** | 176.8 | 57.4 | 29.35 | 8.238 | - | 118.8 |
| **D48** | 176.5 | 54.69 | 40.65 | 8.248 | 4.611 | 118.8 |
| **T49** | 174.3 | 63.19 | 69.44 | 7.853 | 4.119 | 114 |
| **F50** | 175.3 | 58.32 | 38.96 | 8.101 | 4.472 | 120.5 |
| **H51** | 174.4 | 55.77 | 28.94 | 7.907 | 4.62 | 118.3 |
| **I52** | 175.9 | 62.37 | 38.28 | 7.953 | 3.981 | 119.4 |
| **I53** | 176.1 | 61.5 | 37.81 | 7.703 | 4.082 | 119.5 |
| **R54** | 176.6 | 55.88 | 29.79 | 7.891 | 4.316 | 121.1 |
| **K55** | 177.3 | 56.87 | 32.31 | 7.985 | 4.223 | 120.5 |
| **G56** | 174.3 | 45.29 | - | 8.217 | - | 108.8 |
| **S57** | 174.7 | 58.57 | 63.96 | 8.009 | 4.469 | 114.9 |
| **I58** | 176.2 | 61.62 | 38 | 8.008 | 4.184 | 121.1 |
| **S59** | 174.8 | 57.96 | 64.11 | 8.173 | 4.533 | 117.3 |
| **S60** | 174.5 | 58.62 | 63.6 | 8.168 | 4.401 | 117.1 |
| **N61** | 174.9 | 53.58 | 39.06 | 8.169 | 4.652 | 120 |
| **D62** | 176.4 | 54.69 | 40.73 | 8.126 | 4.588 | 119.8 |
| **F63** | 176.3 | 58.53 | 38.92 | 8.057 | 4.529 | 120.4 |
| **E64** | 176.9 | 57.7 | 29.17 | 8.286 | 4.056 | 120.9 |
| **K65** | 177 | 57.01 | 32.24 | 7.865 | 4.194 | 119.4 |
| **S66** | 175.3 | 59.01 | 63.69 | 7.913 | 4.434 | 115.2 |
| **L67** | 178.5 | 57.2 | 41.24 | 8.289 | - | 123.6 |
| **K68** | 177.9 | 58.95 | 31.83 | 7.961 | - | 117.5 |
| **K69** | 177.8 | 57.99 | 32.06 | 7.646 | 4.092 | 118.3 |
| **M70** | 177.4 | 57.39 | 32.75 | 7.908 | 4.257 | 117.7 |
| **C71** | 175.5 | 60.95 | 27.47 | 7.933 | - | 116.6 |
| **K72** | 177 | 57.01 | 31.99 | 7.957 | - | 120.3 |
| **E73** | 177.2 | 56.47 | 29.17 | 8.138 | 4.26 | 118.1 |
| **G74** | 174.6 | 45.56 | - | 8.063 | - | 108.4 |
| **A75** | 178.6 | 53.22 | 18.34 | 8.035 | 4.145 | 122.7 |
| **Y76** | 176.7 | 58.74 | 37.75 | 7.876 | - | 116.8 |
| **W77** | 177.7 | 59.27 | 29.17 | 7.761 | - | 119.9 |
| **G78** | 176 | 46.49 | - | 8.168 | - | 105.3 |
| **A79** | 179.4 | 54.01 | 18.24 | 7.685 | 4.246 | 123.4 |
| **I80** | 177.1 | 63.5 | 37.51 | 7.745 | 3.807 | 117 |
| **A81** | 179.3 | 54.49 | 17.76 | 8.191 | - | 121.8 |
| **G82** | 175.9 | 46.21 | - | 7.927 | - | 103.8 |
| **V83** | 177 | 64.6 | 31.68 | 7.678 | 3.899 | 120.2 |
| **Y84** | 177 | 60.14 | 38.62 | 7.96 | 4.33 | 119.8 |
| **V85** | 177.6 | 64.12 | 31.45 | 8.035 | 3.847 | 117.4 |
| **G86** | 174.9 | 45.7 | - | 7.822 | - | 108.4 |
| **M87** | 176.5 | 56.53 | 32.43 | 7.825 | 4.297 | 119.7 |
| **E88** | 176.7 | 57.27 | 29.05 | 8.167 | - | 119.9 |
| **Y89** | 176.7 | 58.44 | 38.51 | 7.896 | 4.486 | 118.9 |
| **G90** | 174.4 | 45.91 | - | 8.108 | - | 108.5 |
| **V91** | 176.5 | 63.58 | 31.65 | 7.98 | - | 118.9 |
| **E92** | 177.4 | 57.54 | 29.16 | 8.23 | 4.159 | 121.3 |
| **R93** | 177.1 | 56.92 | 29.93 | 7.949 | 4.253 | 119.1 |
| **I94** | 176.9 | 61.93 | 38.11 | 7.891 | - | 118.6 |
| **R95** | 177.1 | 57.31 | 30.03 | 8.101 | 4.277 | 121.4 |
| **G96** | 174.9 | 45.4 | - | 8.194 | - | 108.1 |
| **T97** | 174.7 | 62.46 | 69.63 | 7.894 | 4.285 | 112.4 |
| **R98** | 175.2 | 55.34 | 30.24 | 7.893 | 4.219 | 120.9 |
| **D99** | 176.6 | 53.21 | 40.13 | 8.124 | 4.718 | 120.9 |
| **W100** | 177.2 | 58.69 | 29.03 | 7.858 | 4.342 | 122.7 |
| **K101** | 177.3 | 58.38 | 31.38 | 7.87 | 3.938 | 118.5 |
| **N102** | 175.7 | 53.99 | 38.92 | 7.718 | 4.623 | 116.2 |
| **A103** | 178.1 | 53.03 | 18.62 | 7.803 | 4.277 | 122 |
| **M104** | 176.1 | 56.69 | 32.63 | 7.92 | 4.209 | 116.8 |
| **F105** | 176.9 | 57.42 | 38.41 | 7.824 | 4.806 | 116.5 |
| **G106** | 175.4 | 46.13 | - | 8.138 | - | 108.2 |
| **G107** | 175.4 | 45.33 | - | 8.136 | - | 108.1 |
| **A108** | 179.4 | 54.28 | 19 | 8.04 | 4.228 | 123.4 |
| **V109** | 177.3 | 65.27 | 31.12 | 8.065 | 3.816 | 117 |
| **T110** | 176.9 | 65.2 | 68.45 | 7.982 | 3.9 | 112.4 |
| **G111** | 176.1 | 46.57 | - | 8.171 | - | 108.2 |
| **A112** | 179.9 | 54.42 | 18.17 | 7.726 | 4.214 | 124.4 |
| **L113** | 178.4 | 57.74 | 41.16 | 8.056 | 4.084 | 118.6 |
| **V114** | 178.9 | 65.7 | 31.16 | 8.136 | 3.66 | 117.3 |
| **S115** | 176.5 | 61.07 | 62.98 | 7.988 | 4.206 | 115.5 |
| **A116** | 179.5 | 54.31 | 17.94 | 8.067 | 4.223 | 123.7 |
| **A117** | 178.7 | 53.95 | 18.22 | 8.175 | 4.154 | 119.4 |
| **S118** | 175.4 | 59.74 | 63.6 | 7.984 | 4.322 | 111.8 |
| **N119** | 175.4 | 54 | 38.94 | 7.979 | 4.687 | 118.8 |
| **N120** | 175.1 | 53.86 | 39.12 | 8.029 | 4.737 | 117.7 |
| **K121** | 176.8 | 56.78 | 32.21 | 8.016 | 4.275 | 120.1 |
| **K122** | 176.4 | 56.7 | 32.14 | 8.125 | 4.239 | 120.3 |
| **D123** | 176.1 | 54.65 | 40.64 | 8.09 | - | 119.2 |
| **K124** | 176.4 | 56.25 | 32.69 | 7.959 | 4.314 | 119 |
| **I125** | 175.3 | 61.02 | 38.17 | 7.758 | 4.114 | 119.3 |
| **A126** | 177.6 | 51.7 | 18.78 | 8.032 | 4.523 | 127.2 |
| **V127** | 176.8 | 64.11 | 31.65 | 8.061 | 3.869 | 119.3 |
| **D128** | 177.2 | 55.41 | 40.1 | 8.363 | 4.47 | 119.9 |
| **A129** | 178.7 | 53.33 | 18.83 | 7.759 | 4.282 | 121.8 |
| **I130** | 176.7 | 62.42 | 37.58 | 7.761 | 4.069 | 117.6 |
| **T131** | 176.4 | 63.6 | 69.52 | 8.112 | 4.186 | 113.9 |
| **G132** | 175.8 | 46.38 | - | 8.235 | - | 108.9 |
| **A133** | 179.7 | 54.27 | 18.33 | 7.899 | 4.268 | 123.9 |
| **A134** | 179.1 | 55.06 | 18.12 | 8.341 | 4.123 | 121.9 |
| **I135** | 177.3 | 64.22 | 37.1 | 7.918 | 3.707 | 117 |
| **A136** | 180.7 | 54.72 | 17.83 | 7.852 | 4.183 | 121.1 |
| **T137** | 176.2 | 66.07 | 68.62 | 8.004 | 4.424 | 115.4 |
| **A138** | 178.8 | 55.22 | 17.89 | 8.349 | 4.076 | 123.9 |
| **A139** | 179.9 | 55.07 | 17.75 | 8.489 | 4.028 | 118.5 |
| **E140** | 178.8 | 58.56 | 28.79 | 7.81 | 4.121 | 117.4 |
| **F141** | 177.5 | 60.6 | 39.12 | 8.088 | - | 119.8 |
| **I142** | 178 | 63.99 | 37.1 | 8.373 | 3.748 | 118.3 |
| **N143** | 176.8 | 55.96 | 38.63 | 8.078 | 4.442 | 118.6 |
| **Y144** | 177 | 60.13 | - | 7.921 | 4.309 | 119.1 |
| **L145** | 178 | 56.39 | 41.81 | 7.92 | - | 119 |
| **T146** | 175.4 | 62.91 | 69.43 | 8.001 | 4.217 | 110.9 |
| **L147** | 178 | 55.99 | 41.41 | 7.78 | 4.227 | 122.6 |
| **E148** | 176.7 | 56.59 | 29.51 | 8.014 | 4.132 | 118.1 |
| **H149** | 174.4 | 55.81 | 28.75 | 7.979 | - | 116.9 |
| **H150** | 174.5 | 55.75 | 29.02 | 8.095 | 4.275 | 117.8 |
| **H151** | 174.4 | 55.74 | 29.06 | 8.262 | 4.645 | 118.3 |
| **H152** | 174.1 | 55.58 | 29.11 | 8.233 | 4.647 | 118.5 |
| **H153** | 173.7 | 55.63 | 29.25 | 8.248 | - | 119.1 |
| **H154** | 178.9 | 56.9 | 29.46 | 8.074 | 4.436 | 124.4 |
